# *Hepatozoon canis* in hunting dogs from Southern Italy: distribution and risk factors

**DOI:** 10.1007/s00436-020-06820-2

**Published:** 2020-07-28

**Authors:** L. Pacifico, J. Braff, F. Buono, M. Beall, B. Neola, J. Buch, G. Sgroi, D. Piantedosi, M. Santoro, P. Tyrrell, A. Fioretti, E. B. Breitschwerdt, R. Chandrashekar, V. Veneziano

**Affiliations:** 1 Department of Veterinary Medicine and Animal Productions, Università degli Studi di Napoli “Federico II”, Naples, Italy; 2 IDEXX Laboratories, Inc., Westbrook, ME 04092 USA; 3 Istituto Zooprofilattico Sperimentale del Mezzogiorno, Portici, Italy; 4 Department of Veterinary Medicine, University of Bari Aldo Moro, Valenzano, Italy; 5 Stazione Zoologica Anton Dohrn, Naples, Italy; 6 Comparative Medicine Institute, College of Veterinary Medicine, North Carolina State University, Raleigh, NC 27607 USA; 7 Osservatorio Faunistico Venatorio - Regione Campania, Naples, Italy

**Keywords:** *Hepatozoon canis*, Hunting dogs, Vector-borne diseases, Italy, PCR

## Abstract

*Hepatozoon canis* is a hemoprotozoan organism that infects domestic and wild carnivores throughout much of Europe. The parasite is mainly transmitted through the ingestion of infected ticks containing mature oocysts. The aims of the present survey were to determine the prevalence of *H. canis* in hunting dogs living in Southern Italy and to assess potential infection risk factors. DNA extracted from whole blood samples, collected from 1433 apparently healthy dogs living in the Napoli, Avellino, and Salerno provinces of Campania region (Southern Italy), was tested by a quantitative real-time polymerase chain reaction (qPCR) assay to amplify *H. canis.* Furthermore, the investigated dog population was also screened by qPCR for the presence of *Ehrlichia canis*, a major tick-borne pathogen in Southern Italy, in order to assess possible co-infections. Two hundred dogs were *H. canis* PCR-positive, resulting in an overall prevalence of 14.0% (CI 12.2–15.9). Breed category (*P* < 0.0001), hair coat length (*P* = 0.015), and province of residence (*P* < 0.0001) represented significant risk factors for *H. canis* infection. The presence of *H. canis* DNA was also significantly associated with *E. canis* PCR positivity (*P* < 0.0001). Hunting dogs in Campania region (Southern Italy) are frequently exposed to *H. canis*, and the infection is potentially associated with close contact with wildlife. Further studies are needed to assess the pathogenic potential of *H. canis*, as well as the epidemiological relationships between hunting dogs and wild animal populations sharing the same habitats in Southern Italy.

## Introduction

Canine hepatozoonosis is a vector-borne disease (VBD) caused by hemoprotozoan organisms of the genus *Hepatozoon* (phylum Apicomplexa: Adeleorina), transmitted by ticks (Ixodidae). Currently, two *Hepatozoon* species are known to infect dogs and other wild canids: *Hepatozoon canis* (James 1905) and *Hepatozoon americanum* **(**Vincent-Johnson et al. 1997). *H. canis* is widely distributed in several countries of Europe, Asia, Africa, and America, while *H. americanum* has been reported only from the North American continent (Giannelli et al. 2013; Léveillé et al. 2019). The main vector of *H. canis* is considered to be the brown dog tick, *Rhipicephalus sanguineus* sensu lato (Baneth et al. 2007), and recently, an experimental study has also confirmed the vectorial role of *Rhipicephalus turanicus* (Giannelli et al. 2017); other tick species such as *Amblyomma ovale*, *Haemaphysalis longicornis*, *Haemaphysalis flava*, and *Rhipicephalus (Boophilus) microplus* could be potential vectors of this protozoan parasite (Baneth 2011; de Miranda et al. 2011; Otranto et al. 2011; Orkun and Nalbantoğlu 2018; Léveillé et al. 2019). Transmission to vertebrate hosts occurs through the ingestion of the infected tick vectors, which harbor mature oocysts of *H. canis* (Baneth 2011); after merogonic phase in dog tissues, micromerozoites invade the neutrophils and monocytes, where they mature into gamonts that represent the infective stage for the tick (Baneth et al. 2007). Other routes of infection are the transplacental transmission from the dam to the puppies (Murata et al. 1993). In contrast to *H. americanum*, the transmission by ingestion of *H. canis* monozoic cysts from paratenic host during predation has not been demonstrated (Baneth and Shkap 2003; Baneth 2011).

Based upon *H. canis* epidemiological studies in dogs performed across Europe, the infection prevalence is often correlated to seasonality and the suspected tick vector distribution (Baneth 2011; Otranto et al. 2011; Dantas Torres et al., 2012). Indeed, autochthonous cases were commonly reported where *R. sanguineus* s.l. was endemic (Baneth 2011; Aktas et al. 2015; Ebani et al. 2015; Attipa et al. 2017). However, in recent years, the occurrence of *H. canis* in dogs has been described in areas where *R. sanguineus* s.l. was not found (Hornok et al. 2013; Mitková et al. 2016) and often in association with the presence of *H. canis* in foxes and other wild carnivores (Miterpáková et al. 2017; Hodžić et al. 2018).

Canine hepatozoonosis has generally been characterized as a subclinical infection in dogs. In some cases, infection has been reported in association with clinical signs, such as fever, lethargy, weight loss, and lymphadenomegaly. However, these clinical signs often overlap with those of other diseases (Baneth 2011; Otranto et al. 2011; Giannelli et al. 2013). Furthermore, immunosuppressive chemotherapy or concurrent infections can cause *H. canis* reactivation (Baneth et al. 2003). Immunosuppressed, immunodeficient, and co-infected dogs, in particular, are more likely to develop clinical signs in association with *H. canis* infections (Baneth 2012). Although generally considered an organism of low pathogenicity, rare reports of acute hepatozoonosis, associated with *H. canis*, have been characterized by severe anemia, splenitis, skeletal muscle involvement, and meningoencephalomyelitis (Marchetti et al. 2009).

In Italy, canine hepatozoonosis, associated with *H. canis*, has only been reported in a few clinical case studies or in association with descriptions of diagnostic testing methods (Gavazza et al. 2003; Sasanelli et al. 2009, 2010; Otranto et al. 2011). Large epidemiological surveys involving defined dog populations are sporadic (Cassini et al. 2009; Ebani et al. 2015). As *R. sanguineus* s.l. is the most widespread tick species on the Italian peninsula (Maurelli et al. 2018), hunting dogs may have an increased risk for acquiring *H. canis* due to increased frequency of tick exposure and closer contact with wildlife compared with pet dogs (Piantedosi et al. 2017; Veneziano et al. 2018; Santoro et al. 2019). The aims of this study were to determine the *H. canis* prevalence in hunting dogs living in Southern Italy and to assess the potential risk factors associated with infection. Furthermore, DNA amplification of *Ehrlichia canis*, that is, the most common tick-borne pathogen (TBP) agent in Southern Italy, was obtained in order to verify the possible association with *H. canis* infection, considering that both pathogens can be transmitted by the same tick vector species.

## Material and methods

### Study area

The study was conducted in conjunction with the hunting dog’s health assistance program of University of Naples and was supported by the management committees of the respective hunting districts (ATCs). The region of study encompassed a surface area of 5698.81 km^2^, including the hunting district of Napoli (ATC NA), Avellino (ATC AV), and one of the two hunting districts of Salerno (ATC SA 1). These are located in Southern Italy in the provinces of Napoli (40° 50′ N–14° 15′ E), Avellino (40° 54′ 55″ N–14° 47′ 22″ E), and Salerno (40° 41′ 00″ N–14° 47′ 00″ E). The territory of the three provinces is contiguous, with Napoli and Salerno overlooking the Tyrrhenian Sea. The coastal area has a typical Mediterranean temperate climate that becomes progressively continental in the adjacent inland and mountainous areas.

### Study animals and sample size

A total of 1433 apparently healthy hunting dogs from 153 municipalities representative of the three provinces were included in the study. Between March and November 2015, blood samples were collected by cephalic vein venipuncture from each dog during routine health checks, performed in 44 private veterinary clinics located in the study area. The blood collection did not provide for any segregation or stress of the animal. Each sample was placed in tubes containing potassium ethylene diamine tetra-acetic acid (EDTA), stored at − 80 °C and, defrosted immediately before batch analysis. The study was approved by the Ethical Animal Care and Use Committee of the University of Naples “Federico II” (number of approval 0039904, October 2014). Written informed consent was obtained from the owners of the hunting dogs included in the study.

The necessary sample size to estimate prevalence was calculated using the formula proposed by Thrustfield (1995) considering the following epidemiological data: expected prevalence of 8% for *H. canis* based on the results of a similar study in canine populations from Southern Europe (René-Martellet et al. 2015); confidence interval (99%) and desired absolute precision (2%), based on the number of hunters in Campania region (n° 38,611 hunters in the season 2014–2015 and assuming a dog for each hunter) (BURC 2014).

A questionnaire was submitted to each owner to obtain information about the dog’s residence locality (province), breed category (hound, pointing, mixed-breed), type of coat (short, medium, and long hair), age, gender, pack size when cohabiting with other dogs, contact with other pet or farm animals (dogs, cats, horses, and ruminants), living environment (rural or urban), number of hunting months, type of hunted species (wild mammals or birds), history of tick infestation, and ectoparasite control practices (frequency of ectoparasiticide treatment).

### Molecular assay

*H. canis* and *E. canis* real-time PCR was performed at a commercial laboratory (IDEXX Reference Laboratories, West Sacramento, CA, USA). The target sequences for the *H. canis* and *E. canis* tests were the small subunit ribosomal (*ssr*) and thio-disulfide oxidoreductase (*dsb*) genes, respectively. Briefly, 90 μl of whole blood was resuspended in guanidinium thiocyanate–based lysis solution and incubated for 10 min. Total nucleic acid was isolated on a MagMax96Flex (ThermoFisher) with magnetic beads (Roche Diagnostics) using the manufacturer’s guidelines. Total nucleic acid was eluted in 150 μl of PCR-grade nuclease-free water (ThermoFisher) and 5 μl amplified in subsequent single-plex real-time PCR reactions. Analysis was performed on a Roche Light Cycler 480 (Roche Diagnostics) and raw data analyzed using the 2nd derivative maximum method with the” high sensitivity” setting to generate crossing points (CP values). Real-time PCR was performed in conjunction with six quality controls, including quantitative PCR-positive control, PCR-negative control, negative extraction control, quantitative DNA internal sample quality control targeting the host 18S rRNA gene complex, an internal positive control spiked into the lysis solution, and an environmental contamination monitoring control. All assays were designed and validated according to industry standards (Applied Biosystems 2019).

A subset of 21 *H. canis* and 19 *E. canis* PCR-positive samples were selected for bidirectional Sanger sequencing (University of Delaware DNA Sequencing and Genotyping Center). Amplicons for sequencing were obtained through conventional PCR. The PCR assays consisted of 1× PCR Buffer (Roche Diagnostics), 2.5 mM MgCl_2_ (Roche Diagnostics), 200 μM mixed nucleotides, 2 U ActiTaq exo DNA polymerase (Roche Diagnostics), and 0.5 μM of each primer (TIB MOLBIOL). The cycling profile consisted of 45 cycles of denaturation at 95 °C, annealing at 60 °C, and extension at 72 °C. A 745-bp region of the *H. canis ssr* gene was amplified with primers Hc-sfp (5′ CCG TGG CAG TGA CGG TTA A 3′) and Hc-rfp (5′ GAA GGA GTC GTT TAT AAA GAC GAC CT 3′). For *E. canis*, a 372-bp region of the *dsb* gene was amplified with primers Ec-sfp (5′ GCA AAA TGA TGT CTG AAG ATA TGA AAC A 3′) and Ec-srp (5′ CAC CAC CGA TAA ATG TAT CCC CTA 3′). Sequence homology was determined through BLAST®N analysis (BLASTN 2.9.0+).

### Statistical analysis

To test the effects of risk factors on the probability of testing positive for *H. canis* DNA, a multiple logistic regression was performed. The PCR status (positive vs negative) was considered a response variable, while the risk factors collected on the questionnaire were considered as predictor variables. Proportion positive for *H. canis* DNA was evaluated for each predictor variable, and Clopper-Pearson exact binomial limits were used to determine 95% confidence intervals. Multiple logistic regression was performed using a subset of the predictor variables to determine the odds ratios (OR). Of the 1433 dogs enrolled in the study, 1416 had complete data for the predictor variables and were included in the model. All statistical analyses were performed using SAS software (Version 9.4, SAS Institute Inc., Cary NC) and considering *P* < 0.05 as the threshold for statistical significance. Firth bias-correction was implemented in the multiple logistic regression to address quasi-complete separation due to zero *H. canis* DNA positive dogs from Napoli province.

## Results

**Fig. 1:**
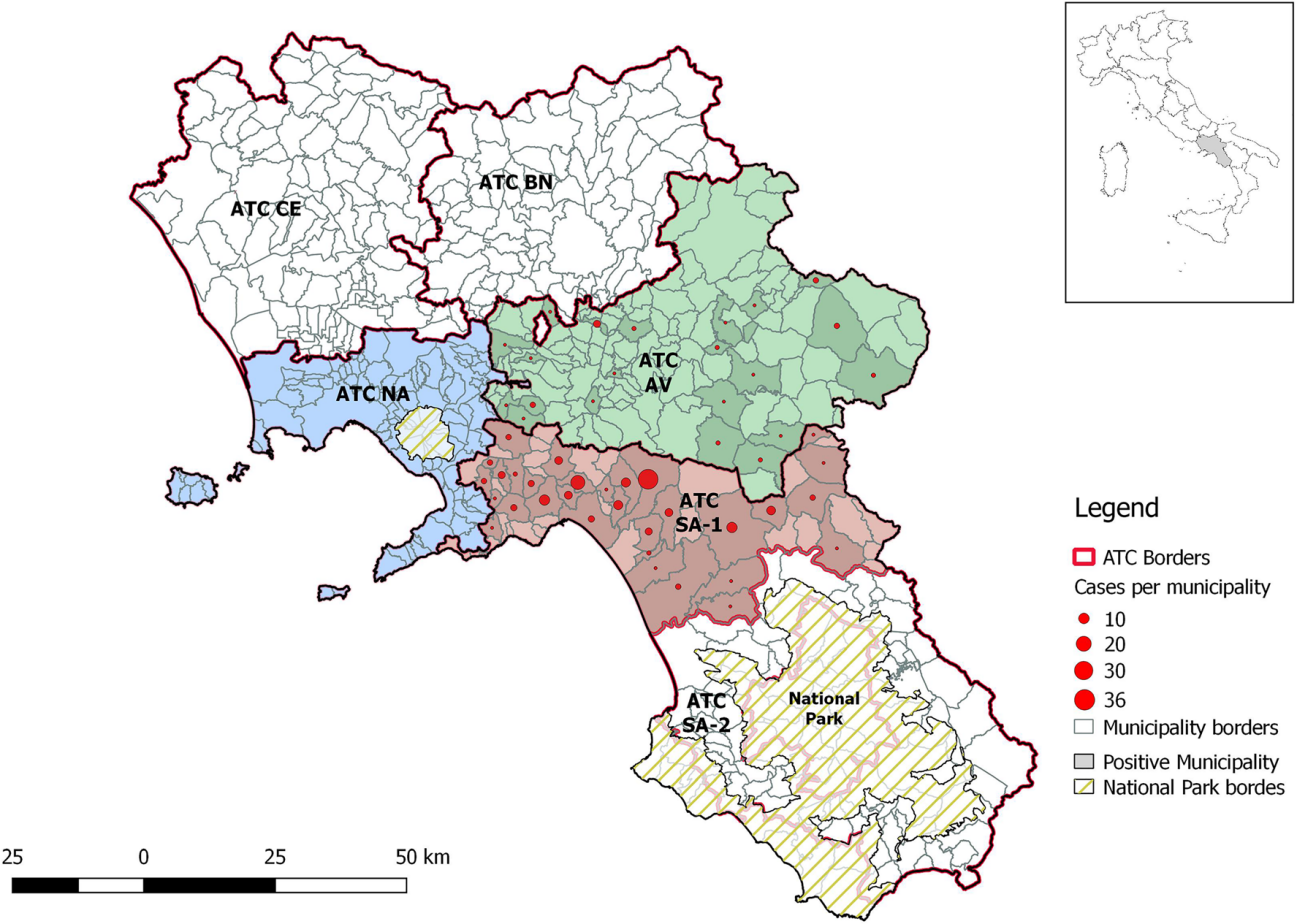
Distribution map of *Hepatozoon canis* PCR-positive hunting dogs in the study area by hunting districts

Two hundred of the 1433 dogs were *H. canis* PCR-positive, with an overall prevalence of 14.0% (200/1433; 95% CI 12.2–15.9%). Thirty-six dogs were PCR-positive to *E. canis* (36/1433; 95% CI 27.9–61.9%) and 16 animals were co-infected by both *H. canis* and *E. canis*. The distribution of the *H. canis* PCR-positive dogs in the study area is shown in Fig. 1. Sequencing of PCR identified *H. canis* as the only species of *Hepatozoon* spp. circulating in dog populations investigated. The sequences showed 99% homology with the corresponding sequence from other dog isolate GenBank sequence MK091085 (*H. canis* isolate 9992-3). The analysis of *E. canis* sequences showed 99–100% homology with GenBank sequences MK783026 (*E. canis* isolate R46) and CP000107 (*E. canis* strain Jake).

The proportions of *H. canis* PCR-positive dogs in relation to the potential risk factors associated with exposure to the parasite are summarized in Table 1. The multiple logistic regression model was developed using risk factors that were expected to be important based upon the biology of the infection or relevant epidemiology. Using a robust model, the presence of *H. canis* DNA was significantly associated with a dog’s breed category (*P* < 0.0001), hair coat length (*P* = 0.015), and living province (*P* < 0.0001); furthermore, a positive correlation was found between *H. canis* and *E. canis* infection (*P* < 0.0001) (Table 1). Risk was higher in dogs with medium (OR 1.89; 95% CI 1.01–3.55) and long hair coat (OR 1.74; 95% CI 1.15–2.62), and in hound breed dogs (OR 1.29; 95% CI 0.64–2.62). Dogs living in Salerno province had the highest risk (OR 5.46; 95% CI 3.56–8.38), while dogs from Napoli area had the lowest risk (OR 0.03; 95% CI 0.00–0.56) for *H. canis* positivity (Table 2). Gender, age, pack size, frequency of ectoparasitic treatment, and tick infestation history were not significantly correlated with *H. canis* infection.

**Table 1:** PCR prevalence (%) and confidence interval (95%) of *H. canis* in hunting dogs in Southern Italy

Variable	Level	Sample*	Prevalence (%) *H. canis* PCR-positive	95% CI
Province	Avellino	552	6.5	4.6–8.9
	Salerno	641	25.6	22.2–29.1
	Napoli	240	0.0	0.0–0.0
Coat	Long	791	12.6	10.4–15.2
	Medium	80	22.5	13.9–33.2
	Short	558	14.7	11.9–17.9
Breed	Hound	525	20.2	16.8–23.9
	Mixed-breed	59	22.0	12.3–34.7
	Other	22	22.7	7.8–45.4
	Pointing	821	9.1	7.3–11.3
Tick infestation history	No	714	12.0	9.7–14.7
	Yes	712	16.0	13.4–18.9
Gender	Female	642	12.2	9.7–14.9
	Male	789	15.5	13–18.2
Living environment	Rural Area	1356	14.5	12.7–16.5
	Urban Area	71	4.2	0.9–11.9
Cohabitation with other pet or farm animals	No	143	13.3	8.2–20
	Yes	1284	14.1	12.2–16.1
Bird hunting	No	611	21.4	18.2–24.9
	Yes	816	8.5	6.6–10.6
Wild mammal hunting	No	813	8.4	6.6–10.5
	Yes	614	21.5	18.3–25
*Ehrlichia canis* PCR result	Negative	1397	13.2	11.4–15.1
	Positive	36	44.4	27.9–61.9

*Totals by category vary due to missing data

**Table 2:** Logistic regression results for the risk factor effect associated with *Hepatozoon canis* positivity for hunting dogs in Southern Italy

Factor	Level	Reference category	Odds ratio (95% CI)	Degrees of freedom	*P* value
Age			1.02 (0.95–1.09)	1	0.5601
Pack size			0.97 (0.94–1.01)	1	0.1244
Ectoparasiticide treatments/year			1.02 (0.98–1.06)	1	0.3733
Province				2	< 0.0001
	Napoli	Avellino	0.03 (0.00–0.56)		
	Salerno	Avellino	5.46 (3.56–8.38)		
Coat				2	0.015
	Long	Short	1.74 (1.15–2.62)		
	Medium	Short	1.89 (1.01–3.55)		
Breed				3	< 0.0001
	Hound	Mixed-breed	1.29 (0.64–2.62)		
	Pointing	Mixed-breed	0.38 (0.18–0.79)		
	Other	Mixed-breed	0.88 (0.26–3.00)		
Gender	Male	Female	1.37 (0.98–1.92)	1	0.0637

## Discussion

Our results indicate that hunting dogs in Southern Italy are frequently infected with *H. canis*. Comparative data for the general dog population of Campania region are not available; however, studies performed in other areas confirm the presence of *H. canis* throughout Italy. In Central-Northern Italy, Cassini et al. (2009) reported a *H. canis* PCR prevalence of 3.63% in kennel and hunting dogs (14/385) in a molecular survey involving vector-borne pathogens (VBPs). Ebani et al. (2015) reported a *H. canis* prevalence of 32.5% (38/117) in hunting dogs from Central Italy. In addition to other possibilities, these discrepancies with respect to our results may be explained by the different sampling periods (Dantas-Torres et al. 2012) and the spread of *R. sanguineus* s.l. in the different areas of the Italian peninsula (Maurelli et al. 2018). In fact, *R. sanguineus* s.l is considered the most prevalent tick species in Southern Italy as there are favorable climatic conditions for its development and survival (Otranto et al. 2014). In a study performed on ticks collected from owned dog populations, the overall prevalence of *R. sanguineus* s.l. in Southern Italy was 36.1% (Maurelli et al. 2018).

The presence and the prevalence of *H. canis* in its major competent vector, *R. sanguineus* s.l., were also investigated in the southern regions of Italian peninsula. In the Apulia region, Ramos et al. (2014) have reported on 1091 off-host ticks, collected from the environment monthly for 1 year, a *H. canis* prevalence of 13.47%. However, in the same area, Dantas-Torres et al. (2012) have showed a lower *H. canis* prevalence of 2.2% in ticks collected from dogs living in a kennel where an outbreak of canine hepatozoonosis was registered. Both studies found that the presence of the pathogen agent in ticks occurred mostly in warmer seasons, suggesting that the infection in dogs could be more noticeable in summer or in autumn (after the peak of tick abundance) and highlighting the importance of vector seasonality in the dynamic of the infection (Dantas-Torres 2010). Despite widespread distribution of *R. sanguineus* s.l. in Campania region, *H. canis* PCR prevalence reported in hunting dogs in this study was found to be lower than in previous reports. A high *H. canis* infection rate (50.6%; 42/83) was reported in dogs from a kennel heavily infested with *R. sanguineus* s.l. in the Apulia region (Otranto et al. 2011). Although these findings are from Southern Italy, they differ from the current study, perhaps due to characteristics of the population in that the current study involved a larger sample size and owned dogs that regularly received ectoparaciticide treatments.

Based upon previous TBP agent studies involving hunting dogs living in the same area of Italy, *H. canis* appears to be the organism with the highest PCR prevalence, and potentially the lowest virulence. For example, Pantchev et al. (2017) in the same area reported a PCR prevalence of 2.4% for *Anaplasma platys* and 1.9% for *E. canis.* Veneziano et al. (2018) reported *Babesia canis* and *Babesia vogeli* PCR prevalences of 0.15% and 1.1%, respectively. The higher prevalence of *H. canis* compared with the other *R. sanguineus* s.l.*–*associated TBP is supported by studies performed in other endemic areas. Ebani et al. (2015) found an *H. canis* PCR infection rate of 32.5% versus 1.7% for *E. canis*. In Turkey, Aktas and Ozubek (2017) reported *H. canis* as the most prevalent hemoprotozoan pathogen (54.3%) followed by *Babesia* spp. (4.6%). In a recent survey performed in Iraq, *H. canis* was the most prevalent VBP in dogs (33%) and wild carnivores (jackals 49.1% and foxes 47.3%) (Otranto et al. 2019). Potentially, the discrepancy between *H. canis* compared with other VBP prevalences may be related to different transmission routes. Dogs could ingest an infected tick from their haircoats while grooming, before the ectoparasite has the chance to take a blood meal and transmit other pathogens. Furthermore, the ingestion of ticks during hunting activities could increase the possibility of developing infection even in the presence of ectoparasite treatments applied to the dog.

An interesting epidemiological aspect is the demonstration of *H. canis* within temperate lineages of *R. sanguineus* s.l., widespread in Mediterranean areas (Demoner et al. 2013; Dantas-Torres and Otranto 2015). It is known that not all lineages of *R. sanguineus* s.l. are competent to host *E. canis*, and the presence of this major VBP correlates to the tropical lineage of *R. sanguineus* (Moraes-Filho et al. 2015). In a study performed in a kennel from the Apulia region with a higher number of dogs positive for *H. canis*, *R. sanguineus* sp. I (temperate lineage) was reported as the competent vector for this pathogen (Otranto et al. 2011; Dantas-Torres and Otranto 2015). Furthermore, Latrofa et al. (2014) showed the presence of *H. canis* in *R. sanguineus* sp. I and sp. III belonged to temperate lineages, while none of ticks belonged to tropical lineage were found positive. In our study, the higher prevalence of *H. canis* compared with other VBPs, as *E. canis*, could be also explained by a different availability of susceptible vectors. However, studies to assess the *R. sanguineus* s.l. lineages in the study area would be necessary to confirm this hypothesis.

In this hunting dog population, breed category was a significant risk factor for acquiring *H. canis* infection. Hounds, particularly when compared with pointing breeds used for hunting birdlife, could have greater *H. canis–*infected tick exposure due to their strict contact with hunted wild mammals. In fact, as reported by other authors (Ebani et al. 2015; Piantedosi et al. 2017), the close contact with wild mammals or bush/woodland, required by this type of hunting, seemingly results in more frequent exposures for hunting dogs to several TBDs. Fighting and/or biting during hunting places the dog at increased risk of ingesting a parasitized tick on the prey or being exposed to ticks that subsequently infest the dogs (Baneth et al., 2011). It is noteworthy that the acquisition of *H. americanum* infection was described also through carnivorism (Baneth 2011), but this potential transmission route is still not demonstrated for *H. canis*, although Baneth and Shkap (2003) reported the presence of *H. canis* monozoic cysts in the spleen of experimentally and naturally infected dogs.

According to previous surveys, there was not an association with gender (Rojas et al. 2014; Lauzi et al. 2016; Aktas et al., 2017; Licari et al. 2017), although male dogs had a slightly increased, but not significant, risk of contracting *H. canis* infection in the present study. Due to their predilection for roaming behavior, male dogs may have higher environmental exposure to TBDs. In agreement with most previous studies, the rate of *H. canis* infection was not significantly associated with age in our hunting dog population (Rojas et al. 2014; Lauzi et al. 2016). In contrast, Aktas et al. (2015) described a higher infection prevalence in adult dogs, possibly related to a longer duration of vector exposure.

Our data indicates a significantly higher prevalence in medium and long hair dogs, because the hard ticks can cling and attach more easily and not be noticed, as previously described (Hornok et al. 2006). Finally, differences in *H. canis* prevalence between the studied areas highlight that geographical effects, including vector density, activity patterns, and other factors, influence a dog’s exposure to tick-borne pathogens. It is important to point out that in the province of Salerno there are large tracks of wooded areas that contain high wildlife densities (Pittiglio et al. 2018). Furthermore, the Salerno province has the highest concentration of boar hunter teams, with an average number of dogs per packs equal to 4.8 dogs (with a maximum of 25 dogs). It is s noteworthy that previous studies from some European countries (Hungary, Czech Republic, Slovakia, and Austria) involving wild fauna have demonstrated a high *H. canis* prevalence in foxes even in the absence of *R. sanguineus* s.l. (Tolnai et al. 2015; Mitková et al. 2016; Mirtepáková et al., 2017; Hodžić et al. 2018). This phenomenon could be explained by the vertical transmission of parasite from female foxes to the offspring (Hodžić et al. 2018). In these countries where the *R. sanguineus* is lacking, it is still unclear whether sharing the territory with foxes could represent a real risk factor for dogs. In our study, living in the more urbanized province of Napoli, where most of the animals are used for bird hunting, has proved to be a protective factor for hunting dogs (0% of 240 dogs; Table 2). Furthermore, the bird hunters of Napoli province had smaller dog packs (only 1 or 2 dogs for each hunter), and the animals received a better routine care, such as a more frequent ectoparasitic treatments. The average number of ectoparasitic treatment months was 9.0, 7.2, and 4.7 for Napoli, Salerno, and Avellino provinces, respectively (Veneziano et al. 2013).

In our study, there was a correlation between PCR amplification of *H. canis* and *E. canis* DNA. Co-infection of *H. canis* with other VBPs is not uncommon (Mundim et al. 2008), and canine ehrlichiosis, associated with *E. canis*, represents the most common TBD in the study area (Piantedosi et al. 2017). *H. canis* gamonts and *E. canis* morulae have been visualized in the same monocyte in a stained blood smear from a dog (Baneth et al. 2015). The presence of *H. canis* might enhance cellular invasion by other VBPs or could potentiate the pathogenicity of other organisms, such as *Leishmania infantum*, *E. canis*, and *Mycoplasma haemocanis* (Baneth et al. 2015; Attipa et al. 2017, 2018). Nevertheless, a clinical association between *H. canis* infection and the worsening of symptoms for pathogenic VBDs (leishmaniosis and ehrlichiosis) has not been confirmed (Mylonakis et al. 2005; Attipa et al. 2018; Baxarias et al. 2018).

## Conclusions

In conclusion, hunting dogs in Southern Italy are exposed to *H. canis* infection. The prevalence of *H. canis* infection is substantially greater than other regional TBPs. Further studies are necessary to better understand the epidemiological and clinical aspects of this protozoan infection among hunting dogs. Moreover, it would be interesting to clarify the parasite transmission modalities related to the relationship between hunting dogs and sympatric wildlife populations.

## Data Availability

The data supporting the conclusions of this article are included within the article.
